# The relationship between agility, linear sprinting, and vertical jumping performance in U-14 and professional senior team sports players

**DOI:** 10.3389/fspor.2024.1385721

**Published:** 2024-04-09

**Authors:** Thordis Gisladottir, Miloš Petrović, Filip Sinković, Dario Novak

**Affiliations:** ^1^ School of Education, Research Center for Sport and Health Sciences, University of Iceland, Reykjavik, Iceland; ^2^ Faculty of Kinesiology, University of Zagreb, Zagreb, Croatia

**Keywords:** correlation, modified agility *T*-test, change of direction speed, countermovement jump, explosive power

## Abstract

**Aim:**

The aim of this paper is to determine the relationship between the modified agility *T*-test (change of direction speed ability), 20-meter sprint test (linear speed ability), and countermovement jump test (vertical jumping performance) in U-14 and professional senior team sports players.

**Methods:**

The sample included 78 (59 female and 19 male) U-14 athletes (age 11.70 ± 1.33 years, height 153.00 ± 12.20 cm and body mass 47.10 ± 11.20 kg) and 43 (18 female and 25 male) senior professional athletes (age 24.80 ± 6.58 years, height 169.00 ± 9.13 cm and body mass 71.20 ± 15.10 kg). Both samples participated in different team sports including basketball, field hockey, and football. Participants underwent a series of tests to assess their speed, change of direction speed, and explosive power. Speed assessments involved 20-meter sprints (sec), while change of direction speed was measured using the modified agility *T*-test (sec). Explosive power was evaluated through countermovement jumps (CMJ), where concentric mean force (N), concentric peak force (N), concentric peak velocity (m/s), eccentric peak force (N), jump height (cm), peak power (W), peak power/BM (W/kg), RSI (m/s) and vertical velocity (m/s) were determined. Pearsońs product moment-correlation coefficient (*r*) served to determine correlations and linear regression was conducted to explain the relationship between the dependent variable (CODS) and independent variables (S20 m and CMJ). The level of statistical significance was set at *p* < 0.05 and the confidence interval was 95%.

**Results:**

The Pearson product-moment correlation analysis in the U-14 athletes indicated no correlation (*r* = 0.11, *p* = 0.34) between the 20-meter linear sprint speed and the modified change of direction *T*-test. Additionally, the results revealed that 4 out of 10 CMJ values showed a significant moderate correlation (*r* = 0.3, *p* < 0.05) between CMJ and the modified change of direction *T*-test. In contrast, senior players exhibited statistically significant correlations in all variables. A significant correlation (*r* = 0.90, *p* = 0.01) was found between 20-meter linear sprint speed and the modified change of direction *T*-test, while CMJ values showed a range of correlations from moderate to large. In both competitive categories, according to the linear regression model, only linear sprint speed over 20-meters significantly explained (*p* < 0.05) the CODS speed ability, while the other CMJ parameters did not reach the significance level (*p* > 0.05).

**Conclusion:**

The study emphasized the influence of explosive power performance (CMJ) and linear speed (S20 m) on agility (CODS) within the sample, particularly among professional senior team sports players. These findings indicate that agility, linear sprinting, and jumping abilities may share common underlying factors.

## 1 Introduction

In team sports, players execute a variety of movements including tackling, blocking, and jumping, whilst also showcasing technical skills (1). Therefore success in these sports depends on a combination of physical, technical, and tactical abilities (2). Recently, there has been an increased focus on the physical demands of team sports due to their evolving nature (3, 4). Speed, agility, and explosive power represent a set of motor abilities important for performance in team sports for both genders (5–7). These abilities are commonly treated together because they use the same energy resources, affect the nervous system similarly, and share common factors influencing their level (8). More precisely, the neuromuscular demands for agility, linear sprinting, and jumping share coordination of muscle actions, the utilization of fast-twitch muscle fibers, and the efficiency of energy transfer (8). It is also believed that athletes with good speed and explosive abilities can better handle intense training and game situations, subsequently reducing the risk of injuries (8). In line with this, improvements, or variations in one aspect (sprint speed or jumping ability), may influence the athlete's agility performance (9, 10).

Agility is considered one of the most important aspects that should be developed in strength and conditioning routines for team sports players (11–14). It is described as the ability to change direction or movement quickly and effectively (12). There are two relatively independent manifest forms of agility: non-reactive or pre-planned change of direction speed (CODS—Change of Direction Speed) and reactive or non-planned agility (RAG—Reactive Agility) (15, 16). CODS involves a rapid change of direction that is pre-planned and known in advance, and players do not need to react to a specific stimulus. Contrary to CODS, RAG incorporates a cognitive aspect, encompassing factors of perception and decision-making (17, 18). Therefore, it can be concluded that RAG manifests in situations where an athlete needs to perform an agile movement but must react to a stimulus (9).

For agility training programs to be optimized, it is essential to establish a correlation analysis with other fitness variables such as power, speed, and strength. Numerous studies have examined the relationship between linear sprinting, change of direction speed, and explosive power abilities in team sports. Alemdaroglu (19) reported significant correlations in a study involving 12 first-division male basketball seniors between the countermovement jump, the squat jump, and the 30 m sprint with the standard test of CODS (*T*-Test). Sisic et al. (20) found a high correlation between horizontal jumping capacity and CODS in 92 elite male juniors basketball players. Similarly, Spiteri et al. (21) identified significant correlations between maximal dynamic, isometric, concentric, and eccentric strength with CODS in 12 elite senior female basketball players. It was also established in a study by Pehar et al. (22), that horizontal jump performance (Broad jump test) was found to be an important predictor of basketball-specific CODS in 88 high-level male basketball players. However, Little and Williams (23) indicated no correlation between acceleration speed and maximal speed with agility in 106 male junior Australian soccer players. This observation was consistent with the findings of Young et al. (24) in both Australian soccer and Australian football players. A weak correlation between jumping abilities and reactive agility performance was noted in male junior Australian footballers by Henry et al. (25). In contrast, Spaniol et al. (26) discovered a significant correlation between the 40-yard dash test and the 20-yard shuttle test when examining the correlation between speed and change of direction speed in professional male football players. A study conducted by Križaj (27) with a sample of 18 elite female football athletes also showed that correlation analysis indicated a moderate relationship between CMJ and CODS performance and a very significant relationship between S20 m and CODS performance. Conlon et al. (28) identified a significant relationship between the vertical jump test and agility test performances across 134 male and female professional senior athletes in various sports. Additionally, Pereira et al. (29) concluded a significant correlation between agility and the explosive power of the lower limb in elite female adolescent handball players. Studies have also explored the relationship between speed, agility, and explosive power in elite male rugby players (30–33), hockey players (34), and female softball players (35).

However, it should be noted that there are inconsistencies in these results. While some research indicates strong correlations between these parameters, others suggest weaker relationships. Differences in test results can have specific causes; perhaps they are based on different diagnostic approaches and research procedures, different sports disciplines, or different age categories of the selected investigated samples. In line with this, it can be concluded that correlations between speed, agility, and explosive power vary across different sports and also among different levels of participants. It is evident that professional senior athletes with a higher qualitative and experienced level achieve a high correlation between speed and explosive properties, in contrast to elite junior players where slightly weaker correlations in the mentioned motor abilities were observed. One of the main problems is that there is a lack of research comparing the correlations between agility and other motor components among young athletes and professional senior athletes. Most studies focus only on junior or senior categories, even though it is known that biological age and maturity status have a significant effect on the motor performance of youth athletes (36–38). Because of this, the expected correlation between agility, speed, and explosive power is higher among senior athletes than among youth athletes.

In accordance with the above, the aim of this paper was to determine the relationship between the modified agility *T*-test (change of direction speed ability), 20-meter sprint test (linear speed ability), and countermovement jump test (vertical jumping performance) in U-14 and professional senior team sports players. It was hypothesized that agility, sprint time, and jumping ability would be influenced by each other and would collectively indicate an athlete's overall fitness capability, particularly among professional senior team sports players.

## 2 Methods

### 2.1 Participants

The sample included 78 (59 female and 19 male) U-14 athletes (age 11.70 ± 1.33 years, height 153.00 ± 12.20 cm and body mass 47.10 ± 11.20 kg) and 43 (18 female and 25 male) senior professional athletes (age 24.80 ± 6.58 years, height 169.00 ± 9.13 cm and body mass 71.20 ± 15.10 kg). Both samples participated in different team sports, including basketball, field hockey, and football. The G-Power program (version 3.1.9.2; Heinrich Heine University, Dusseldorf, Germany) was used to estimate the required number of participants, considering an expected effect power of *f* = 0.33, an alpha level of 0.05, and a statistical power of 0.90. To be eligible for participation in the study, all participants had to meet specific inclusion criteria, including being physically active players who trained for at least six hours per week and participated in regional, national, or international tournaments. More precisely, all U-14 participants were actively engaged in their respective national top division leagues tailored to their age cohorts, while senior participants encompass both professional athletes from the realm of football and top-tier players in field hockey, amalgamating a diverse pool of athletic expertise. Exclusion criteria encompassed any injury that would impact play and physical performance at the study's commencement. The study was conducted in accordance with the Helsinki Declaration and approved by the Ethics Committee of the University of Iceland (SHV2023-078 and SHV2023-048). All participants were informed about the research's purpose and the conditions for participation. Both participants and their parents provided prior written consent to participate. The complete testing protocol was thoroughly explained to them, with particular emphasis on the additional effort required for the research and the risk of injury, which was equivalent to the risk during standard training or competition.

### 2.2 Measurements and procedure

The sample for anthropometric variables in this study included measurements of participant height and body mass. Body height (cm) was measured using a portable altimeter (Seca 213; Seca GmbH, Hamburg, Germany), and body mass (kg) was measured by standing on two force platforms (VALD performance, ForceDecks Mini, Brisbane, Queensland, Australia). Participants underwent a series of tests to assess their speed, change of direction speed, and explosive power. Speed assessments involved 20-meter sprints (sec), while change of direction speed was measured using the modified agility *T*-test (sec). Explosive power was evaluated through countermovement jumps (CMJ), where concentric mean force (N), concentric peak force (N), concentric peak velocity (m/s), eccentric peak force (N), jump height (cm), peak power (W), peak power/BM (W/kg), RSI (m/s) and vertical velocity (m/s) were determined. The Powertimer system (Newtest Oy, Oulu, Finland) was utilized for measuring speed and change of direction speed, while the VALD performance force plates were used to assess explosive power during jumps. Each test was performed three times, and the average of the three trials was calculated for further analysis.

Before the testing session, all participants underwent a standardized warm-up tailored to team sports. The warm-up comprised several activities, starting with light-intensity running, covering a distance of 10 sets of 20 meters each. Subsequent to the running component, participants engaged in dynamic stretching exercises, lasting a total of 15 min. These dynamic stretches incorporated lateral movements, skipping, jumping, lunges, and concluded with four repetitions of sub-maximum acceleration.

#### 2.2.1 Linear sprint speed test

For the linear sprint speed test, two photocells were positioned at the start location and at the 20 m distance marker. Participants were instructed to adopt their preferred foot positioning, placed on a marked line on the floor, and initiate the sprint from a stationary standing start. The time was recorded from passing the first photocells and until the sprinters crossed the 20 m finish line as quickly as possible. Each participant performed three trials, with a 3 to 4 min rest period between each trial. The mean value of the three trials was calculated for further analysis (9).

#### 2.2.2 Explosive power test

During the countermovement jump test, participants maintained holding their hands on their hips to minimize any influence from the upper body on jump performance. Starting from a standing position with their knees straight, participants executed a squat motion, lowering themselves to approximately a 90° angle, and then rapidly accelerated in a vertical direction using both legs. Each participant completed three trials of the tests, with a 1 min rest period between each trial. The mean value of the three trials was then calculated for further analysis. Through the analysis of the jumps, values were obtained for concentric mean force (N), concentric peak force (N), concentric peak velocity (m/s), eccentric peak force (N), jump height (cm), peak power (W), peak power/BM (W/kg), RSI (m/s) and vertical velocity (m/s) (9).

#### 2.2.3 Change of direction speed test

The modified agility *T*-test was conducted using the same protocol as the standard *T*-test, with the exception of modifications to the total distance covered and measures of inter-cone distance (39, 40). However, the number of directional changes remained consistent. In the modified *T*-test, participants covered a total distance of 20 meters, as opposed to 36.56 meters in the standard *T*-test. The criteria for accepted test trials remained the same as those used in the standard *T*-test. For the modified *T*-test setup, a configuration of four cones was arranged in the shape of a “T”. The starting cone was positioned 5 meters away from the first cone, whilst two additional cones were placed 2.5 meters away on either side of the second cone. Participants were instructed to sprint from the start line to the first cone (5 meters) and touch it with their right hand. They then shuffled 2.5 meters to the left to reach the second cone, touching it with their left hand. Afterward, they shuffled 5 meters to the right to reach the third cone, touching it with their right hand, and subsequently moved 2.5 meters back to the middle cone, touching it with their left hand. Finally, participants ran backward as quickly as possible to return to the start line. Timing began with a signal and ended when the participant crossed the timing gate upon their return. Trials were deemed unsuccessful if participants failed to touch a designated cone, crossed their legs during shuffling, or did not face forward throughout the test. Timing was recorded in hundredths of a second.

### 2.3 Statistical analysis

The obtained data was processed in the program Jamovi (version 2.3.28). The normality of the distribution was tested with the Shapiro–Wilk W test. Basic descriptive parameters (mean—xˉ; standard deviation—SD) were used to describe each variable. Pearsońs product moment-correlation coefficient (*r*) served to determine correlations. A threshold of 0.1 was defined as small, between 0.3 and 0.5 as moderate, between 0.5 and 0.7 as large, between 0.7 and 0.9 as very large, and above 0.90 as extremely large correlations (41). Linear regression was conducted to explain the relationship between the dependent variable (CODS) and independent variables (S20 m and CMJ). The level of statistical significance was set at *p* < 0.05 and the confidence interval was 95%.

## 3 Results

The results of demographic and anthropometric descriptive statistics for the U-14 participants and the senior professional athletes are presented in Table 1.

**Table 1:** Descriptive statistics of the U-14 and senior professional sample.

	Sport	*N*	Gender	Age [years]	Body height [cm]	Body weight [kg]
				(Mean ± *SD*)	(Mean ± *SD*)	(Mean ± *SD*)
Senior group	Field hockey	11	Male	29.30 ± 8.90	176.0 ± 5.60	77.20 ± 15.40
		18	Female	23.60 ± 5.60	161.00 ± 6.00	65.40 ± 15.20
	Football	14	Male	22.90 ± 3.50	175.00 ± 6.30	74.00 ± 13.00
U-14 group	Basketball	59	Female	12.40 ± 1.10	156.00 ± 10.80	51.20 ± 14.70
	Football	19	Male	10.00 ± 0.70	139.00 ± 5.20	37.70 ± 5.30

SD, standard deviation.

The results of the Pearson product-moment correlation analysis in U-14 athletes showed that there is no correlation (*r* = 0.11, *p* = 0.34) between 20-meter linear sprint speed and the modified change of direction *T*-test. Also, results showed that there is no correlation (*r* < 0.3, *p* > 0.05) between CMJ values for concentric mean force, concentric peak force, eccentric peak force, jump height, peak power, and the modified change of direction *T*-test. However, some of the results showed that there was a significant moderate correlation (*r* = 0.3, *p* < 0.05) between CMJ values for concentric peak velocity, peak power/BM, RSI, vertical velocity and the modified change of direction *T*-test (Table 2).

**Table 2:** Results of Pearson's correlation analysis (*r*) in the U-14 sample.

Variables	S20 m	CMJ (concentric mean force)	CMJ (concentric peak force)	CMJ (concentric peak velocity)	CMJ (eccentric peak force)	CMJ (jump height)	CMJ (peak power)	CMJ (peak power/BM)	CMJ (RSI)	CMJ (vertical velocity)
CODS (*T*-TEST)	*r* = 0.11	*r* = −0.19	*r* = −0.09	*r* = −0.30	*r* = −0.01	*r* = −0.16	*r* = −0.17	*r* = −0.30	*r* = −0.30	*r* = −0.35
	*p* = 0.34	*p* = 0.10	*p* = 0.41	*p* = 0.01*	*p* = 0.93	*p* = 0.17	*p* = 0.14	*p* = 0.02*	*p* = 0.02*	*p* = 0.01*

CODS, change of direction speed ability (agility *T*-TEST); S20 m, 20 m linear sprint; CMJ, countermovement jump without arm swing.

* Significant at the level of *p *< 0.05.

In contrast to the U-14 sample, where the correlation proved significant in 4 out of 10 measured variables, in senior players, statistically significant correlations were obtained in all variables. The results showed a statistically very large correlation (*r* = 0.90, *p* = 0.01) between 20-meter linear speed and the modified change of direction *T*-test. Furthermore, a significant moderate correlation (*r* = 0.3–0.5, *p* = 0.01) was observed between CMJ values for concentric mean force, concentric peak force, and eccentric peak force. Additionally, for CMJ values including concentric peak velocity, jump height, peak power, peak power/BM, RSI, and vertical velocity, a large correlation (*r* = 0.5–0.8, *p* = 0.01) with the modified change of direction *T*-test was obtained (Table 3).

**Table 3:** Results of Pearson's correlation analysis (*r*) in the senior professional sample.

Variables	S20 m	CMJ (concentric mean force)	CMJ (concentric peak force)	CMJ (concentric peak velocity)	CMJ (eccentric peak force)	CMJ (jump height)	CMJ (peak power)	CMJ (peak power/BM)	CMJ (RSI)	CMJ (vertical velocity)
CODS (*T*-TEST)	*r* = 0.90	*r* = −0.46	*r* = −0.51	*r* = −0.64	*r* = 0.40	*r* = −0.81	*r* = −0.73	*r* = −0.69	*r* = −0.58	*r* = −0.65
	*p* = 0.01*	*p* = 0.01*	*p* = 0.01*	*p* = 0.01*	*p* = 0.01*	*p* = 0.01*	*p* = 0.01*	*p* = 0.01*	*p* = 0.01*	*p* = 0.01*

CODS, change of direction speed ability (agility *T-*TEST); S20 m, 20 m linear sprint; CMJ, countermovement jump without arm swing.

* Significant at the level of *p *< 0.05.

In the linear regression model (Table 4), only 20-meter linear sprint speed significantly explained (*p* = 0.02) the CODS speed ability, while the other CMJ parameters did not reach the significance level (*p* > 0.05) for the junior U-14 sample.

**Table 4:** Effects of independent variables on the dependent variable in the linear regression model in the U-14 sample.

DV	IV	B	*SE*	*β*	*t*	*p*
CODS	S20 m	3.90	1.66	0.39	2.33	0.02*
	CMJ (cmf)	0.00	0.01	0.45	0.68	0.50
	CMJ (cpf)	−0.01	0.01	−0.99	−1.34	0.18
	CMJ (cpv)	−3.47	2.32	−0.77	−1.50	0.14
	CMJ (epf)	−4.44	0.00	−0.06	−0.12	0.90
	CMJ (jh)	0.20	0.12	0.31	1.72	0.09
	CMJ (pp)	0.00	0.00	0.66	0.93	0.35
	CMJ (ppB)	−0.08	0.05	−0.53	−1.50	0.14
	CMJ (rsi)	−3.27	3.28	−0.17	−1.00	0.32
	CMJ (vv)	2.71	1.93	0.68	1.41	0.17

DV, dependent variable; IV, independent variables; B, unstandardized coefficient; *SE*, standard error, standardized regression coefficient; *t*, statistic; *p*, significance level; CODS, change of direction speed ability (agility *T-*TEST to the left and right); S20 m, 20 m linear sprint; CMJ, countermovement jump without arm swing; cmf, concentric mean force; cpf, concentric peak force; cpv, concentric peak velocity; epf, eccentric peak force; jh, jump height; pp, peak power; ppb, peak power/BM; rsi, RSI; vv, vertical velocity.

* Significant at the level of *p *< 0.05.

Similar to junior athletes, in the linear regression model (Table 5), only 20-meter linear sprint speed significantly explained (*p* = 0.01) the CODS speed ability, while the other CMJ parameters did not reach the significance level (*p* > 0.05).

**Table 5:** The effects of the independent variables on the dependent variable in the linear regression model in the senior professional sample.

DV	IV	B	*SE*	*β*	*t*	*p*
CODS	S20 m	3.59	0.59	0.80	6.13	0.01*
	CMJ (cmf)	−1.30	0.00	−0.02	−0.06	0.96
	CMJ (cpf)	−5.38	0.00	−0.11	−0.34	0.73
	CMJ (cpv)	−10.63	5.62	−2.48	−1.89	0.07
	CMJ (epf)	−2.84	0.00	−0.06	−0.17	0.87
	CMJ (jh)	−0.01	0.04	−0.04	−0.18	0.86
	CMJ (pp)	0.00	0.00	0.01	0.03	0.97
	CMJ (ppB)	−0.02	0.06	−0.15	−0.35	0.73
	CMJ (rsi)	−0.27	1.19	−0.03	−0.22	0.82
	CMJ (vv)	10.48	5.53	2.56	1.90	0.07

DV, dependent variable; IV, independent variables; B, unstandardized coefficient; SE, standard error, standardized regression coefficient; *t*, statistic; *p*, significance level; CODS, change of direction speed ability (agility *T-*TEST to the left and right); S20 m, 20 m linear sprint; CMJ, countermovement jump without arm swing; cmf, concentric mean force; cpf, concentric peak force; cpv, concentric peak velocity; epf, eccentric peak force; jh, jump height; pp, peak power; ppb, peak power/BM; rsi, RSI; vv, vertical velocity.

* Significant at the level of *p *< 0.05.

## 4 Discussion

The main purpose of the study was to investigate the relationship between the results of the modified agility *T*-test (CODS), 20-meter linear sprint speed, and different values of the countermovement (CMJ) test. The results indicated that the correlation analysis in U-14 athletes showed there was no statistically significant correlation between 20-meter linear sprint speed and the modified change of direction *T*-test. Additionally, the results revealed that 4 out of 10 CMJ variables showed a significant correlation between CMJ and the modified change of direction *T*-test. In contrast, senior players exhibited statistically significant correlations in all variables. These findings indicate that agility, linear sprinting, and jumping abilities may share common underlying factors. Additionally, the hypothesis that the relationship between agility, linear sprinting, and vertical jumping performance would be higher among professional senior team sports players has been confirmed. The differences between U-14 and professional senior players are also evident in the range of correlation. Based on the obtained results in U-14 players, it can be noticed that the correlations marked as significant are not extremely large, ranging within 0.30–0.35, indicating a moderate correlation. On the other hand, correlations in senior players mostly vary between large and very large, ranging from 0.60–0.90. More specifically, the largest correlation (*r* = 0.90) was obtained between the 20-meter linear sprint speed, which is logical since this test contains the most similar movement patterns to the modified agility *T*-test. Furthermore, the results of this study showed that in both competitive categories, according to the linear regression model, only 20-meter linear sprint speed significantly explained (*p* < 0.05) the CODS speed ability, whilst the other CMJ parameters did not reach the significance level (*p* > 0.05). Based on the presented results, it appears that professional senior athletes with a higher qualitative and experienced level achieve a high correlation between speed and explosive properties, in contrast to elite junior players where slightly weaker correlations in the mentioned motor abilities were observed. However, this does not necessarily mean that tests should not be performed in young age groups. The decision to conduct testing in younger age groups should consider various factors, including the specific testing goals and potential benefits and risks. In line with that, while the relationship may be more pronounced in senior players, testing in young age groups should not be dismissed but should be tailored to their abilities and objectives.

When comparing the results of this study with previous research, it can be determined that the obtained values are in line with expectations. In a study investigating 12 first-division male seniors, Alemdaroglu (19) reported significant correlations between the countermovement jump (*r* = −0.59) and the 30 m sprint (*r* = 0.50) with a standard test of CODS (*T*-Test). Despite the obtained statistically significant correlation, it is considered moderate but not large. The study conducted by Križaj (27) with a sample of 18 elite female football athletes (18.56 ± 2.24 years) showed that correlation analysis indicated a moderate relationship between CMJ and CODS performance (*r* = –0.59, *p* = 0.01) and a very significant relationship between S20 m and CODS performance (*r* = 0.80, *p* = 0.00). The results of linear regression proved that both independent variables (S20 m and CMJ) explained about 60.0% of the total model variance in relation to the dependent variable (CODS), whereas only S20 m significantly (*p* < 0.05) explained CODS ability. Additionally, Sisic et al. (20) reported a large correlation between jumping capacity and CODS in 92 elite male juniors, whilst in a study by Spiteri et al. (21), significant correlations between maximal dynamic, isometric, concentric, and eccentric strength with CODS (*r* = −0.79 to −0.89) were found in 12 elite female junior basketball players. Furthermore, an investigation of youth elite basketball players of both genders revealed that unilateral jump performance and body mass were the most important correlates with CODS for boys, and sprint performance was strongly correlated with CODS in girls (74.8% of the variance) (42). Moreover, Little and Williams (23) reported no correlation between acceleration speed and maximal speed with CODS in the group of 106 professional male Australian soccer players. Similar results were obtained by Young et al. (24), where no significant correlation was shown between straight sprinting tests and CODS tests in either Australian soccer or Australian football players. However, in line with the results, regarding Australian footballers, researchers found a weak correlation between vertical jumping abilities and agility performance (25). Spaniol et al. (26) investigated the correlation between speed and change of direction speed of professional male football players and found a significant correlation between the 40-yard dash test and the 20-yard shuttle test. Also, Conlon et al. (28) found a significant relation between the vertical jump test and agility test performances in 134 professional athletes from differing sports. Findings of their study have shown that vertical jump velocity is the strongest determinant of speed and CODS performance. A significant correlation between CODS and explosive power of the lower limb in elite adolescent handball players was concluded by Pereira et al. (29). What is also important to emphasize is that neuromuscular training could potentially improve agility performances. More precisely, in recent years, several systematic reviews and meta-analyses have focused on the effect of plyometric training on athletic performance in various team sports, including volleyball, soccer, and basketball (43–45). The results have shown that, in most cases, neuromuscular training positively influences the development of agility, but differently in male and female athletes. There are also a number of studies that focus on establishing the correlation between speed, agility, and explosive power in samples of elite junior rugby players (30–33) as well as in samples of hockey players (34), and female softball players (35). The observed differences in the mentioned studies, especially between male and female athletes and from differing sports, can be attributed to several factors. Firstly, inherent physiological differences between the genders, such as muscle mass, hormonal profiles, and biomechanics may influence how individuals perform in agility, sprinting, and jumping tasks. Additionally, different movement patterns and skill demands in various sports may contribute to variations in the relationship between speed, agility, and explosive power. For example, team sports such as basketball, field hockey, and football require different sets of motor skills, and athletes may prioritize certain aspects based on positional requirements. The nature of these sports, combined with individual playing styles, could explain the variability in the correlation between linear sprint speed, change of direction speed, and explosive power in athlete samples. Understanding these nuances is critical for tailoring training interventions and performance assessments to meet the specific needs of athletes in different sports and genders.

This study has certain limitations that should be considered. Firstly, the study primarily focused on team sports and samples such as U-14 players and professional seniors, raising questions about the generalizability of the results to individual sports. Additionally, differences in training, experience, and techniques among participants should be considered. For example, professional seniors may have more advanced technical skills or deeper tactical knowledge, which could influence their performance in agility and sprint tests. In line with that, the quality of participants' movements, which could impact their performance, was not evaluated. In future research, it is recommended to involve expert and licensed coaches to assess participants' movement techniques during test performance to evaluate the effect of this aspect on the results. It is also essential to consider that the choice of tests for assessing motor abilities can affect the study outcomes. Different tests might yield varying results and introduce a degree of bias based on test selection. In line with this future research would benefit from the use of more specific reactive agility tests. The results of this study can assist coaches in creating personalized development plans for their athletes, considering their individual strengths and weaknesses in the context of agility, speed, and jumping performance. Additionally, the findings of this study provide coaches with valuable insights for devising training methods aimed at enhancing agility, speed, and explosive power. However, future studies (i.e., clinical outcome research) should provide more information on whether the change observed in a group of patients who received the intervention with the average change observed in patients who received no intervention was meaningful or important as opposed to a trivial difference. This would provide additional information and will help coaches to assess and decide upon the results.

## 5 Conclusions

The results of this research indicate that the correlation analysis in U-14 athletes showed no correlation between 20-meter linear sprint speed and the modified change of direction *T*-test. Additionally, the results revealed that 4 out of 10 CMJ variables showed a significant moderate correlation between CMJ and the modified change of direction *T*-test. In contrast, senior players exhibited statistically significant correlations in all variables. Consequently, it can be said that the study emphasized the influence of explosive power performance (CMJ) and linear speed (S20 m) on agility (CODS) within this sample, particularly among professional senior team sports players. These findings offer valuable insights for coaches in designing diverse sport specific scenarios to enhance overall performance, particularly focusing on the neuromuscular fitness of their players.

## Data Availability

The raw data supporting the conclusions of this article will be made available by the authors, without undue reservation.
